# Bioactive Glass Modified by Sonochemistry Improves Peri-Implant Bone Repair in Ovariectomized Rats

**DOI:** 10.3390/biomimetics10120821

**Published:** 2025-12-08

**Authors:** Marcelly Braga Gomes, Nathália Dantas Duarte, Gabriel Mulinari-Santos, Fábio Roberto de Souza Batista, Luy de Abreu Costa, Paulo Roberto Botacin, Paulo Noronha Lisboa-Filho, Roberta Okamoto

**Affiliations:** 1 Department of Basic Sciences, School of Dentistry (FOA-UNESP), São Paulo State University “Júlio de Mesquita Filho”, Araçatuba 16015-050, SP, Brazil; marcelly.braga@unesp.br (M.B.G.); luy.abreu-costa@unesp.br (L.d.A.C.); paulo.botacin@unesp.br (P.R.B.); 2 Department of Diagnosis and Surgery, School of Dentistry (FOA-UNESP), São Paulo State University “Júlio de Mesquita Filho”, Araçatuba 16015-050, SP, Brazil; nd.duarte@unesp.br (N.D.D.); fabio.rs.batista@unesp.br (F.R.d.S.B.); 3 Division of Restorative and Prosthetic Dentistry, College of Dentistry, The Ohio State University, Columbus, OH 43210, USA; 4 Department of Physics, School of Sciences (FC-UNESP), São Paulo State University “Júlio de Mesquita Filho”, Bauru 17033-360, SP, Brazil; paulo.lisboa@unesp.br

**Keywords:** biocompatible materials, bone regeneration, dental implants, sonochemistry, sonochemical technique, osseointegration, osteoporosis

## Abstract

Estrogen deficiency is a primary cause of osteoporosis, compromising bone mineral density that may impair peri-implant healing. Given the compromised bone environment associated with estrogen deficiency, strategies such as particle reduction via sonochemistry are promising approaches to enhance regenerative outcomes. However, its effects in promoting bone formation remain insufficiently explored. Therefore, this study evaluated the potential of two sonicated biomaterials to improve peri-implant repair in ovariectomized rats. Fifty female rats were allocated into five groups: blood clot (CLOT), Biogran^®^ (BGN), sonicated Biogran^®^ (BGS), Bio-Oss^®^ (BON), and sonicated Bio-Oss^®^ (BOS). Tibial peri-implant defects were created 30 days after ovariectomy and analyzed 28 days later by removal torque, microcomputed tomography, and confocal microscopy. BGS exhibited the highest removal torque (6.28 Ncm), followed by BON (5.37 Ncm), BOS (3.92 Ncm), BGN (3.15 Ncm), and CLOT (2.58 Ncm). Micro-CT revealed bone volume fraction (BV/TV) values of 8.07% (CLOT), 6.47% (BOS), 6.02% (BGS), 5.55% (BGN), and 2.84% (BON). For the trabecular number (Tb.N), BGS (1.11 mm^−1^) showed a significant increase compared with BGN (0.69 mm^−1^), *p* < 0.05. These findings show that sonochemically modified bioactive glass improves mechanical stability and trabecular microarchitecture under estrogen-deficient conditions. However, further studies are needed to standardize sonication parameters for different biomaterials and expand their translational applicability.

## 1. Introduction

Estrogen deficiency is one of the primary causes of osteoporosis, and its early stage—osteopenia—compromises bone microarchitecture, reduces bone mass and mechanical strength, and may impair peri-implant healing [1,2]. Although previous studies have not reported a direct inhibition of osseointegration, this systemic condition is consistently associated with lower primary implant stability in osteoporotic bone and an increased risk of implant failure [3,4,5]. This underscores the clinical need for biomaterials that enhance regenerative potential under estrogen-deficient systemic conditions, particularly in postmenopausal patients, who represent a substantial proportion of candidates for oral rehabilitation with dental implants [5].

Women in early menopause also exhibit marked elevations in RANKL, expressed by osteoblasts, bone marrow stromal cells, and immune cells such as T and B lymphocytes, which binds to its receptor RANK on osteoclast precursors to drive differentiation, multinucleation, activation, and bone resorption [6]. Thus, postmenopausal osteopenia arises from a profound imbalance between osteoblastic bone formation and osteoclastic resorption, predominantly mediated by reduced estrogen levels [7]. In preclinical studies, this systemic condition is commonly reproduced by bilateral ovariectomy in Wistar rats, a validated model for inducing osteopenia [8,9].

Although the osteogenic efficacy of clinically available biomaterials is generally satisfactory, their osteoconductivity can be further optimized through sonochemistry [10,11]. This technique uses ultrasonic waves in a liquid medium to fragment microscale particles, thereby increasing the surface area available for biological interactions [10,12]. The resulting larger surface area improves the material’s physicochemical properties and enhances osteoconduction by providing more sites for protein adsorption, cell adhesion, and initial matrix deposition [13]. These modifications enhance the biomaterial’s biological responsiveness and strengthen cell–biomaterial interactions, enabling the biomaterial to function more effectively as a scaffold for new bone formation [14]. Furthermore, sonochemistry is recognized as a “green technology” because it often uses less energy, requires no toxic chemicals, and generates less waste, enabling rapid, efficient surface modifications, particularly compared with traditional chemical methods [15,16].

Recent studies have primarily used sonochemistry to homogenize drugs with biomaterials [9,17,18]. However, the current problem lies in the scarcity of literature applying this technique as a strategy to reduce particle size, thereby improving the performance of commercial biomaterials for bone formation, particularly under osteopenic conditions. Therefore, the novelty of the present study is to explore the potential of sonochemistry as a particle-reduction method to enhance the osteoconductive properties of two distinct commercial biomaterials, Biogran^®^ (Biomet 3i Innovations Inc., Palm Beach Gardens, FL, USA), a silica-based bioactive glass [18], and Bio-Oss^®^ (Geistlich, Wolhusen, Switzerland), a heterogeneous bovine-derived xenograft composed of inorganic bovine granules [19], in ovariectomized rats, used here as a peri-implant bone defect model under estrogen-deficient conditions, providing a biologically challenging environment for the tested biomaterials.

## 2. Materials and Methods

### 2.1. Ethics and Sample Size

After approval by the Ethics Committee on Animal Experimentation under protocol No. 0499-2022, the study was initiated. Fifty adult female Wistar rats, six months old, and weighing an average of 300 g, were obtained from the Central Animal Facility of FOA-UNESP. Animals were kept under controlled conditions with a 12 h light/dark cycle, receiving water and a balanced diet with 1.4% calcium and 0.8% phosphate (Nuvilab, Curitiba, PR, Brazil). Additionally, this study was conducted in accordance with the ARRIVE Guidelines [20]. Animals were randomly assigned to experimental groups, and calibrated examiners performed all analyses under blinded conditions to reduce bias. The sample size for this study was calculated using a power test (OpenEpi, Version 3, Open-Source Calculator), based on previously published results with a similar methodology using removal torque values [18]. Therefore, the number of animals per group was 10.

### 2.2. Groups

The rats were divided into five groups (n = 10): CLOT, BGN, BGS, BON, BOS. In the CLOT group, the peri-implant bone defects were filled only with spontaneous blood clots. In the other groups, after ovariectomy, peri-implant bone defects were created and implants were placed with commercial biomaterials: Biogran^®^ in natura (BGN), sonicated Biogran^®^ (BGS), Bio-Oss^®^ in natura (BON), and sonicated Bio-Oss^®^ (BOS).

### 2.3. Estrous Cycle

Rats were kept individually in the animal facility for two weeks, after which estrous cycles were monitored for an additional two weeks. Only rats with standard cycling patterns were selected for the experiment. Vaginal smears were obtained by introducing two drops of saline, which were aspirated and placed on histological slides for microscopic evaluation, to identify the phases of the estrous cycle, as described [21].

### 2.4. Ovariectomy

According to studies, bilateral ovariectomy induces progressive bone alterations, with osteopenia developing within 30 days in Wistar rats [9,22]. Experimental models confirm that estrogen deficiency compromises bone repair [23]. Animals underwent a 12 h preoperative fast and were sedated intramuscularly with Ketamine 70 mg/kg and Xylazine 6 mg/kg. A strict aseptic protocol was followed, including the use of sterilized instruments, sterile drapes, gowns, and gloves.

Trichotomy of the abdominal region was performed, followed by antisepsis with a povidone-iodine detergent (PVPI 10%). A midline abdominal incision, slightly to the right, was made with a blade on a scalpel handle. After entering the peritoneal cavity, adipose tissue was retracted to expose the uterine tube and ovary, which were exteriorized. The surgical wound was closed with simple sutures. Thirty days after ovariectomy, the estrous cycle was evaluated to confirm its absence, ensuring surgical success and induction of osteopenia. After confirmation, peri-implant bone defects were created, and implants were placed.

### 2.5. Biomaterials Modified by Sonochemistry

The reduction of the materials was performed at the Advanced Materials and Nanotechnology Laboratory (FC-UNESP) (Principal Investigator: P.N.L-F). Samples of Biogran^®^ and Bio-Oss^®^ were homogenized by sonochemistry using a beaker with 20 mL of ultrapure water (Milli-Q^®^; Millipore, Burlington, MA, USA) to achieve uniform humidity and reduce particle size. The use of Milli-Q^®^ water increased solute vapor in cavitation bubbles, enhancing acoustic cavitation effects.

A preliminary study tested different sonication times with Biogran^®^, showing that 15 min of sonication provided the best performance. Sonicated Biogran^®^ demonstrated improved extracellular matrix properties, higher bone quality and quantity, and better tissue turnover in peri-implant repair. Thus, a 15 min protocol was standardized for further research [11]. Samples were processed in a Sonics^®^ VCX-750 (Sonics & Materials, Newtown, CT, USA; 750 W, 20 kHz) for 15 min at 40% amplitude. During synthesis, the chamber was kept under atmospheric conditions, with the container and ultrasonic tip cooled in an ice bath. Samples were identified as BGS and BOS and subsequently dried in an oven at 70 °C. Finally, all samples were standardized to 25 mg, the volume of biomaterial used to fill each peri-implant defect, and sterilized using UV light before the implant surgery for 20 min.

### 2.6. Implant Placement

The animals were fasted for 8 h before the surgical procedure and sedated by intramuscular injection with a combination of Ketamine 70 mg/kg and Xylazine 6 mg/kg. After sedation, trichotomy was performed on the medial portion of the right and left tibiae, and the surgical area was disinfected with PVPI 10%. Surgeries were performed in a randomized distribution.

**Figure 1:**
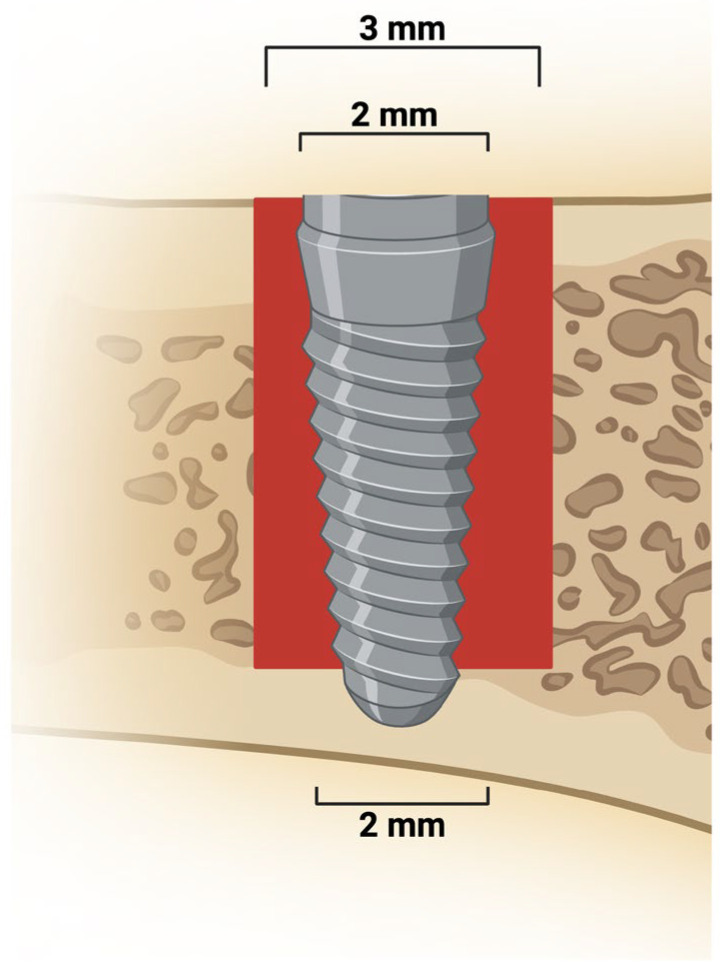
Peri-implant bone defect and implant placement. Representative image shows a 3 mm defect extending from the upper cortical to the medullary bone. A 2 mm diameter implant was placed and stabilized in the lower cortical bone. Created in BioRender. Duarte, N. (2025) https://BioRender.com/s3fm8mm.

An incision of approximately 1.5 cm was made on the left and right tibial metaphysis. The soft tissue was thoroughly dissected and retracted with periosteal elevators to expose the bone. Osteotomy was performed using a 1.3 mm spiral drill through both cortices, followed by a 2.0 drill and a 3.0 mm drill in the upper cortical and medullary bone of the tibiae, mounted on an electric motor (BLM 600^®^; Driller, São Paulo, SP, Brazil) at 1000 rpm, under irrigation with 0.9% sodium chloride solution, using a contra-angle handpiece with 20:1 reduction (Handpiece 3624N 1:4; Head 67RIC 1:4; KaVo^®^, Biberach, Germany). A total of 80 commercially pure grade IV titanium implants (Titaniumfix^®^, São José dos Campos, SP, Brazil), with double acid-etched surfaces (nitric, hydrofluoric, and sulfuric acids), measuring 2 mm in diameter and 4 mm in height (2 × 4 mm), sterilized by gamma radiation, were fixed in the lower cortical, as observed in Figure 1. The peri-implant defects were filled with the biomaterials specific to each experimental group, except in the CLOT group. The volume of biomaterial used to fill the peri-implant defects corresponded to the defect created by the 3 mm diameter drill to a depth of 3.5 mm (volume = 12.56 mm^3^), minus the volume of the implant measuring 1.5 mm × 3.5 mm (volume = 6.18 mm^3^), totaling 18.56 mm^3^ of biomaterial to ensure biomaterial-implant contact along the entire implant surface. The volume was calculated using the formula: V = π·r^2^·h (volume equals Pi times radius squared times height). Each animal received two implants, one in each tibial metaphysis. Tissues were sutured in layers, with continuous sutures in the deep layer and interrupted sutures in the superficial layer. Postoperatively, each animal received a single intramuscular dose of 0.2 mL of penicillin G benzathine. The animals were kept in cages with five animals each, with food and water ad libitum.

### 2.7. Fluorochromes

Calcein (20 mg/kg) and alizarin (30 mg/kg) were injected intramuscularly at 14 and 24 days postoperatively, respectively. Calcein indicates calcium deposition in old bone, and alizarin indicates new bone [24].

### 2.8. Euthanasia

**Figure 2:**
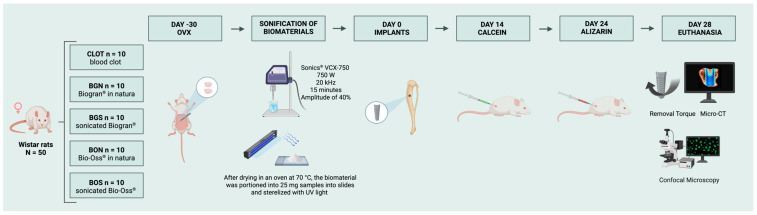
Experimental groups and timeline. At day-30, ovariectomy (OVX) was performed to induce osteopenia. The sonicated biomaterials were prepared before surgery. At day 0, thirty days after ovariectomy, peri-implant bone defects were created and filled with the designated biomaterials, except in the CLOT group, and titanium implants were subsequently placed. Calcein and alizarin fluorochromes were administered 14 and 24 days post-surgery, respectively. Euthanasia occurred at 28 days, followed by removal torque, micro-CT, and confocal microscopy. Created in BioRender. Duarte, N. (2025) https://BioRender.com/dfltvjk.

After 28 days post-implant surgery, euthanasia was performed via intraperitoneal overdose of 2.5% sodium thiopental 150 mg/kg and 2% lidocaine 10 mg/kg. The tibia of five animals per group was dissected for removal torque analysis. The tibia of the remaining animals per group was collected and stored in 70% ethanol for microcomputed tomography and confocal microscopy. The overview of the experimental groups and the timeline is illustrated in Figure 2.

### 2.9. Biomechanical Analysis

The tibias were accessed to expose the implants and perform reverse torque measurements. An implant driver was fitted to the implant hexagon, and a digital torque meter was attached to the driver. In the right tibial metaphysis, a counterclockwise rotation was applied, gradually increasing the reverse torque until the implant rotated within the bone, completely disrupting the bone-implant interface. At this point, the torque meter recorded the maximum torque at failure, expressed in Newton per centimeter (Ncm).

### 2.10. Micro-CT

After euthanasia, the left tibias were reduced and fixed in 10% buffered formalin solution for 48 h and rinsed in running water for 24 h. After fixation, the samples were stored in 70% ethanol for micro-CT using Skyscan 1272 (Bruker, Aatselaar, Belgium) at the Multi-User Laboratory of the FOA-UNESP. Images were captured with a camera pixel size of 12.45 mm, at a resolution of 2672 × 4000 (rows per scan), with 8 μm thick slices, a 90 kV X-ray beam, 111 μA current, a 0.5 mm Al filter, and a 0.4° rotation step. The images were then reconstructed using NRecon (SkyScan; Bruker, Aatselaar, Belgium) with smoothing set to 1, ring artefact correction of 3, beam hardening correction of 5%, and image correction variation from 0.0 to 0.11. In the Data Viewer (SkyScan; Bruker, Aatselaar, Belgium), images were aligned, and a new dataset was saved. Analyses were performed using CTAn (SkyScan; Bruker, Aatselaar, Belgium) to evaluate 100 slices counted from the lower bone cortical. This analysis provided mineral density values and a three-dimensional characterization of the bone tissue formed around the implants and in the peri-implant repair area. The evaluated parameters included bone volume (BV, mm^3^), bone volume per tissue volume (BV/TV, %), trabecular number (Tb.N, mm^−1^), trabecular thickness (Tb.Th, mm), trabecular separation (Tb.Sp, mm), and intersection surface (IS, mm^3^). Finally, the three-dimensional reconstruction of the peri-implant bone area was performed using CTvox (SkyScan; Bruker, Aartselaar, Belgium).

### 2.11. Confocal Microscopy

After processing the scanned samples for micro-CT analysis, slides were prepared to assess bone tissue dynamics through calcein and alizarin fluorochromes. Samples were processed for calcified sections, embedded in methyl methacrylate, and polished manually until 150 μm thick sections were obtained. The samples were analyzed by laser confocal microscopy to evaluate fluorochrome labelling. Longitudinal sections of the peri-implant defect regions were captured using a Stellaris 5 confocal microscope (Leica Microsystems, Heidelberg, Germany) with a 10× objective (100× total magnification). The separate images of calcein (green) and alizarin (red) were reconstructed to provide an overlay of the fluorochromes. Bone dynamics were analyzed using ImageJ, version 1.54p (Madison, WI, USA). The daily mineral apposition rate (MAR, μm) was calculated by measuring the distance between calcein and alizarin labels along a line perpendicular to the labels, then dividing this measurement by 10 to account for the interval in days between the two fluorochrome injections. This analysis allows the evaluation of MAR on the bone formed around implants.

### 2.12. Statistical Analyse

Data normality was assessed with the Shapiro–Wilk test (*p* > 0.05). For normally distributed data, a parametric test, one-way ANOVA, was applied to all analyses, followed by Tukey’s post hoc test for multiple comparisons using SigmaPlot (Version 10; Systat Software Inc., San Jose, CA, USA). Differences were considered significant at *p* < 0.05.

## 3. Results

The overall quantitative data from the study are summarized in Table 1 and detailed below.

### 3.1. Removal Torque

**Figure 3:**
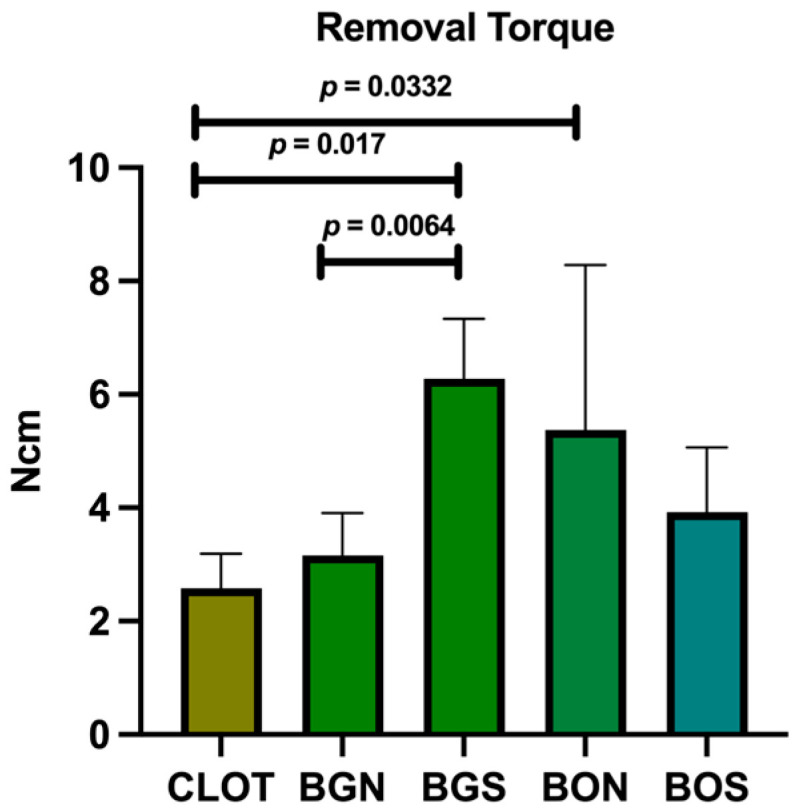
Biomechanical test of removal torque. Statistically significant differences are indicated by bars with *p*-value, determined using one-way ANOVA followed by Tukey’s test (*p* < 0.05).

The implants were successfully placed, and their stability was confirmed by biomechanical testing. As shown in Figure 3, the BGS group had the highest mean removal torque (6.28 Ncm), which was significantly higher than that of CLOT and BGN. Moreover, CLOT showed a considerably lower value compared to BON.

### 3.2. Micro-CT

**Figure 4:**
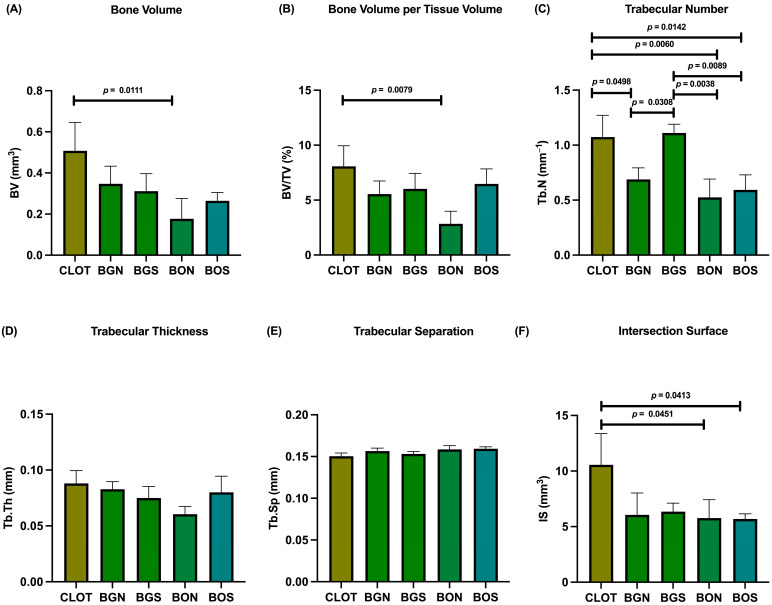
Micro-CT of peri-implant bone. The parameters were evaluated as follows: (**A**) bone volume (BV); (**B**) bone volume per tissue volume (BV/TV); (**C**) trabecular number (Tb.N); (**D**) trabecular thickness (Tb.Th); (**E**) trabecular separation (Tb.Sp); (**F**) intersection surface (IS). Statistically significant differences are indicated by bars with *p*-value, determined using one-way ANOVA followed by Tukey’s test (*p* < 0.05).

**Figure 5:**
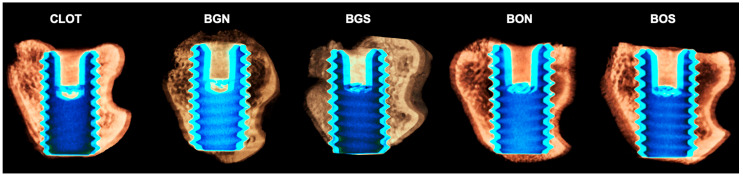
Representative three-dimensional images of the peri-implant bone area of all five groups: CLOT, BGN, BGS, BON, and BOS, respectively. It is possible to observe an increased bone around implant threads in the medullary compartment of the BGS and BOS groups, beyond the CLOT group. Scale is not applicable for volumetric renderings generated in CTvox software, version 2.7.

In the evaluation of bone volume fraction, the following results were obtained for the CLOT, BGN, BGS, BON, and BOS groups: 8.07%, 5.55%, 6.02%, 2.84%, and 6.47%, respectively. Therefore, BV and BV/TV showed a significant difference between CLOT and BON (Figure 4A,B). For Tb.N, significant differences were found between CLOT and BGN, CLOT and BON, CLOT and BOS, BGN and BGS, BON and BGS, and BGS and BOS (Figure 4C). In addition, Tb.Th and Tb.Sp did not differ significantly among the groups (*p* > 0.05; Figure 4D,E). Regarding IS, significant differences were detected between CLOT and BON and between CLOT and BOS (Figure 4F). Representative three-dimensional images of peri-implant bone around the installed implants in all five groups are shown in Figure 5.

### 3.3. Confocal Microscopy

**Figure 6:**
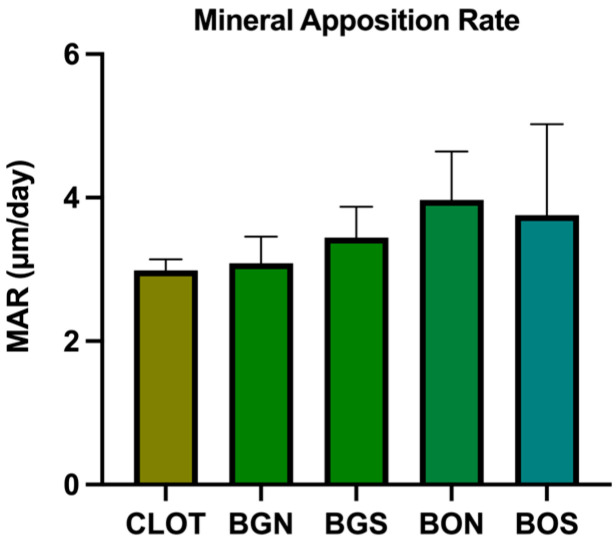
Confocal microscopy, mineral apposition rate (MAR). No statistically significant difference was found between the groups (one-way ANOVA; *p* > 0.05).

**Figure 7:**
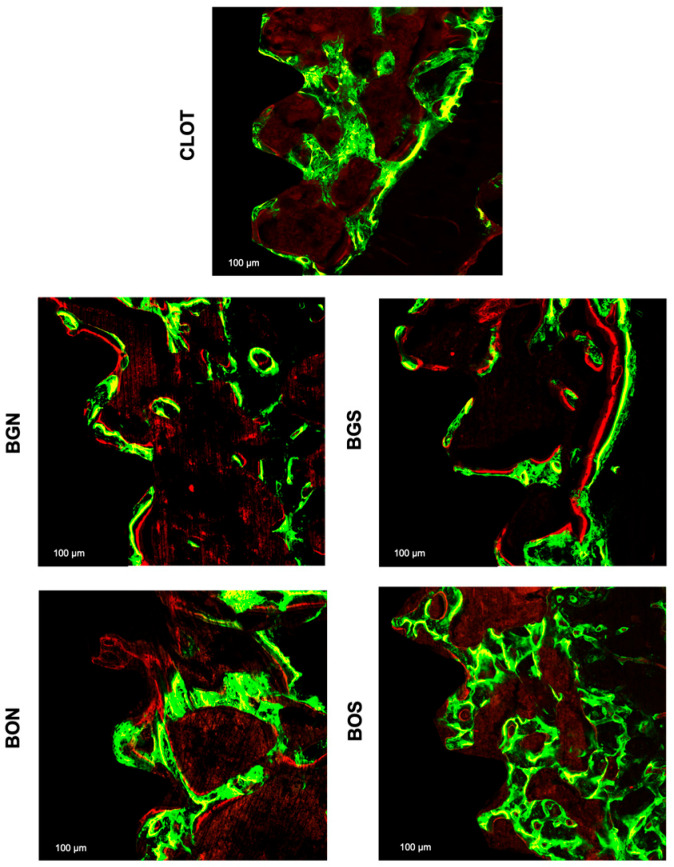
Representative fluorescence images of bone dynamics between the third and fifth medullary implant threads with a scale of 100 µm in all groups: CLOT, BGN, BGS, BON, and BOS. The green fluorochrome calcein labelled old bone was applied at 14 days, and the red fluorochrome alizarin labelled new bone at 24 days.

For MAR, no statistically significant difference was found between the groups (*p* > 0.05), as observed in Figure 6. Representative images of overlapped fluorochromes for each group are shown in Figure 7.

## 4. Discussion

In clinical scenarios, many patients with insufficient bone quality or quantity for implant placement frequently present with bone resorption and structural defects [5]. This represents a primary challenge in individuals with compromised skeletal conditions, such as osteopenia or osteoporosis [2]. To address these deficiencies, biomaterials have been employed to enhance the predictability of oral implant rehabilitation [25]. However, biomaterials still exhibit performance limitations that warrant further optimization, and sonochemistry has emerged as a promising technique [12].

In the present study, sonicated biomaterials showed potential to enhance peri-implant bone repair, improving both biomechanical stability and trabecular microarchitecture. Specifically, the BGS group demonstrated superior biomechanical strength, suggesting that sonochemistry may enhance the physicochemical properties of the bioactive glass biomaterial, thereby increasing surface reactivity and promoting stronger bone-to-implant interfacial stability, which is essential for osseointegration [26]. Biogran^®^, when treated with sonochemistry, increases the exchange of sodium, calcium, and hydrogen ions, forming an external layer of silica and hydroxyapatite that promotes bone formation [11]. These improved extracellular matrix properties, increased bone quantity and quality, and enhanced bone repair have also been reported in other studies [27]. It is important to note that the 15 min sonochemistry time was standardized based on a previous study that reported the largest bone formation area of 18,409.74 μm^2^ and a MAR of 3.7 μm/day in the sonicated Biogran^®^ group [11].

However, the biological relevance of BGS and its clinical significance in relation to natural bone still need to be investigated. On the other hand, BOS promoted better trabecular organization without showing increased mechanical stability, perhaps due to its inherent porosity or slower resorption kinetics. The divergent responses between Biogran^®^ and Bio-Oss^®^ highlight that the effectiveness of sonochemistry is material-dependent, influenced by intrinsic composition, mineral crystallinity, and ion-release profiles.

Micro-CT analysis corroborated these biomechanical results, demonstrating significant differences in bone parameters among the groups. Interestingly, the CLOT group exhibited favorable BV, BV/TV, and IS, reinforcing the intrinsic osteogenic potential for bone repair. The blood clot provides a vital matrix enriched with platelets, cytokines, and growth factors that can assist in bone repair in minor or four-wall bone defects [28,29]. In line with this, previous studies have shown that clot-derived signaling contributes to bone repair, although the absence of biomaterials may compromise bone stability and mechanical integrity [30]. In clinical scenarios, bone resorption during healing may lead to alveolar ridge defects and insufficient implant support [31]. Biomaterials play a critical role as space maintainers, preventing bone defects and preserving bone architecture while new bone gradually replaces and integrates with the biomaterial [32]. Thus, although blood clots exhibit intrinsic osteogenic potential, biomaterials are essential primarily for ensuring mechanical stability, especially in larger or compromised bone defects [32].

Furthermore, Tb.Th and Tb.Sp did not differ significantly among groups, suggesting that both parameters were preserved, a finding fundamental to maintaining functional trabecular bone architecture. The MAR from confocal microscopy also did not differ statistically among groups. However, in the qualitative comparison, calcein green labeling was more intense than alizarin red, suggesting that bone formation was more pronounced in the early phase at 14 days than in the late phase at 24 days. The short period between alizarin injection and euthanasia may have limited detection of late mineralization, which has also been previously reported [33].

Curiously, BOS also showed favorable outcomes in some microarchitectural parameters. Although it did not reach the highest biomechanical stability, the BOS group showed a higher Tb.N trend, similar to the BGS group, compared with its in natura form. This suggests that sonochemistry enhanced interconnected trabecular bone compared to the other biomaterials. Additionally, the tendency toward improvement in BV/TV and Tb.Th comparison with the in natura form reinforces the role of sonochemistry in optimizing microstructural organization.

Other studies confirm the results for Bio-Oss^®^. In its in natura form, Bio-Oss^®^ has demonstrated bone formation in direct contact with the biomaterial, despite a lower percentage of bone formation (16.1%); it retained more biomaterial (37.2%), potentially increasing implant stability [34]. Another study found similar bone formation between in natura and sonicated Bio-Oss^®^, but the residual biomaterial was 34.62 ± 3.54 for the in natura form and 20.50 ± 4.50 for the sonicated form [17]. Previous RT-PCR analysis also indicated greater bone remodeling with sonicated Bio-Oss^®^, with higher gene expression of RANKL, osteoprotegerin, osteocalcin, and bone sialoprotein compared with the other experimental groups [9].

Relative to other particle-reduction techniques, sonochemistry offers distinct advantages. Traditional approaches such as ball milling, air-jet micronization, and cryomilling are effective for reducing particle size [35,36,37]. However, they often induce undesirable alterations, including contamination by milling media, disruption of crystallinity, amorphization, and reduction in surface porosity, factors known to reduce biomaterial bioactivity [38]. In contrast, sonochemical processing relies on acoustic cavitation, which promotes particle refinement, increases surface area, largely preserves the material’s chemical structure, and enhances ionic reactivity [12,14,16]. Thus, sonochemistry appears to provide a superior balance between particle modification and preservation of physicochemical integrity, contributing to enhanced biological and mechanical outcomes.

Despite the promising results of this study, several limitations must be acknowledged. The primary limitation is the absence of morphological analyses, such as scanning electron microscopy and quantitative particle-size assessment by laser diffraction, which limits the mechanistic interpretation of how particle reduction contributed to the observed outcomes. Another limitation is the inability to histologically evaluate biomaterial behavior, as undecalcified sections were not available. Additionally, the sonication time was based on a single previous study using Biogran^®^ [11], and alternative durations were not evaluated for other biomaterials. Ultrasonic frequency also affects sonochemical reduction rates [30]. However, only 20 kHz was used in this study. Moreover, the long-bone model does not fully capture the dynamics of alveolar bone healing [39]. Lastly, there is a lack of analysis of inflammatory markers, which could have provided additional insight into differences in immune responses among the biomaterials and the blood clot. Future investigations should incorporate detailed physical characterization of particle modifications induced by sonochemistry, along with standardized protocols, to better elucidate the physical and biological mechanisms underlying the clinical applicability of sonicated biomaterials.

Clinically, this study has significant implications and market potential, enabling rapid, safe, effective, and user-friendly modification of biomaterials for clinical and industrial applications. Furthermore, it is economically viable and sustainable [14,16], making it attractive for large-scale production if sonication time, temperature, and energy consumption are optimized. However, confirmation of these benefits requires standardized protocols and additional clinical studies to validate the efficacy observed in other experimental models.

## 5. Conclusions

Sonicated Biogran^®^ demonstrated superior biomechanical stability and improved trabecular organization, as evidenced by a higher trabecular number, suggesting that this technique enhances its osteoconductive properties. Although Bio-Oss^®^ also showed modest improvements after sonication, these effects were less pronounced. Overall, the findings support that sonochemistry is a simple and effective strategy to optimize bioactive glass for peri-implant bone repair. However, further studies are needed to standardize sonication parameters for different biomaterials and expand their translational applicability.

## Figures and Tables

**Table 1:** Mean and standard deviation (Mean ± SD) for all groups in each analysis: biomechanical test (removal torque), micro-CT (BV, BV/TV, Tb.N, Tb.Th, Tb.Sp, IS), confocal microscopy (MAR).

Analysis	CLOT	BGN	BGS	BON	BOS
Removal Torque (Ncm)	2.583 ± 0.6113	3.157 ± 0.7525	6.28 ± 1.055	5.375 ± 2.909	3.925 ± 1.141
BV (mm^3^)	0.5077 ± 0.1376	0.3472 ± 0.08601	0.3112 ± 0.08537	0.177 ± 0.09877	0.2643 ± 0.0408
BV/TV (%)	8.07 ± 1.873	5.548 ± 1.197	6.023 ± 1.418	2.836 ± 1.146	6.471 ± 1.373
Tb.N (mm^−1^)	1.074 ± 0.1974	0.688 ± 0.1054	1.11 ± 0.08016	0.5251 ± 0.1666	0.593 ± 0.1364
Tb.Th (mm)	0.08802 ± 0.01146	0.08287 ± 0.006735	0.07492 ± 0.01027	0.06051 ± 0.00692	0.08001 ± 0.01446
Tb.Sp (mm)	0.1504 ± 0.003897	0.1565 ± 0.003615	0.1531 ± 0.00302	0.1586 ± 0.004437	0.1594 ± 0.002245
IS (mm^3^)	10.56 ± 2.812	6.063 ± 1.964	6.352 ± 0.7552	5.764 ± 1.663	5.683 ± 0.4703
MAR (μm)	2.987 ± 0.1514	3.087 ± 0.3711	3.443 ± 0.4291	3.97 ± 0.6736	3.757 ± 1.266

## Data Availability

The authors will make the raw data supporting this article’s conclusions available upon request.

## Abbreviations

The following abbreviations are used in this manuscript:

ARRIVE	Animal Research: Reporting of In Vivo Experiments
BGN	Biogran^®^ in natura
BGS	Biogran^®^ sonicated
BON	Bio-Oss^®^ in natura
BOS	Bio-Oss^®^ sonicated
BV	Bone volume
BV/TV	Bone volume per tissue volume
CEUA	Animal Ethics Committee
FC-UNESP	Bauru School of Sciences—São Paulo State University
FOA-UNESP	Araçatuba School of Dentistry—São Paulo State University
IBSP	Integrin binding sialoprotein
LSMT	Laboratory for Study of Mineralized Tissue
MAR	Mineral Apposition Rate
micro-CT	Microcomputed tomography
OVX	Ovariectomy
OCN	Osteocalcin
OPG	Osteoprotegerin
RANK	Receptor activator of nuclear factor kappa-Β
RANKL	Receptor activator of nuclear factor kappa-Β ligand
RT-PCR	Real Time Reverse Transcription Polymerase Chain Reaction
SD	Standard deviation
Tb.N	Trabecular number
Tb.Sp	Trabecular separation
Tb.Th	Trabecular thickness
IS	Intersection surface
%	Percentage
°C	Degrees Celsius
g	Gram
h	Hour(s)
kg	Kilogram
mg	Milligram
mg/kg	Milligram per kilogram
mL	Milliliter
mm	Millimeter
mm^−1^	Per millimeter
mm^3^	Cubic millimeter
μm	Micrometer
μm^2^	Square micrometer
μm/day	Micrometer per day
Ncm	Newton centimeter
rpm	Revolutions per minute
